# A Comparison of Microtensile Bond Strength, Film Thickness, and Microhardness of Photo-Polymerized Luting Composites †

**DOI:** 10.3390/ma15093050

**Published:** 2022-04-22

**Authors:** Farid El-Askary, Abdullah Hassanein, Emad Aboalazm, Nadin Al-Haj Husain, Mutlu Özcan

**Affiliations:** 1 Operative Dentistry Department, Faculty of Dentistry, Ain Shams University, Cairo 11566, Egypt; 2 Conservative Dentistry Department, Faculty of Oral and Dental Medicine, Egyptian-Russian University, Cairo 11829, Egypt; abdullahhassanien@dent.asu.edu.eg (A.H.); emad_aboalazm73@yahoo.com (E.A.); 3 Division of Dental Biomaterials, Clinic for Reconstructive Dentistry, Center of Dental Medicine, University of Zurich, 8032 Zurich, Switzerland; nadin.al-haj-husain@zmk.unibe.ch (N.A.-H.H.); mutluozcan@hotmail.com (M.Ö.); 4 Department of Reconstructive Dentistry and Gerodontology, School of Dental Medicine, University of Bern, 3008 Bern, Switzerland

**Keywords:** CAD/CAM composite, photo-polymerized composite, dual-cured resin cement, pre-heating, µ-tensile bond strength, film thickness, microhardness

## Abstract

The aim of this study was to evaluate the effect of CAD/CAM composite thickness on micro-tensile bond strength (µTBS), microhardness (HV), and film thickness (FT) of different luting composites. Composite blocks (6.8 mm × 6.8 mm) were divided into 12 groups according to: CAD/CAM thickness and luting composite. For each group, 21 rods (1 mm × 1 mm) were tested in tension at crosshead speed of 1 mm/min. Fracture modes were categorized as adhesive, mixed, and cohesive. Microhardness (*n* = 5/group) was assessed using microhardness tester. Film thickness (12-rods/group) was evaluated using a stereomicroscope (×40). Data were analyzed using the two-way ANOVA/Tukey’s HSD test (*p* = 0.05). Parameters “thickness”, “cement”, and “thickness x cement” showed significant difference on µTBS and HV (*p* < 0.05). At 2 mm, heated *x-tra* fil composite showed the highest µTBS (45.0 ± 8.5 MPa), while at 4 mm thickness, Grandio Flow revealed the lowest µTBS (33.3 ± 6.3 MPa). Adhesive, mixed, and cohesive failures were reported. The HV of all composites decreased when photo-polymerized through 4 mm thickness (*p* < 0.05). Regardless of CAD/CAM thickness, photo-polymerized composites can be successfully used for luting CAD/CAM composite.

## 1. Introduction

Recently, with the introduction of digital technology in the field of dentistry, computer-aided design/computer-aided manufacturing (CAD/CAM) technology has become a trend in the restorative dentistry daily clinical work. In massively destructed teeth, due to caries or failure of existing restorations, direct restorations are considered time consuming, requiring operator skills to obtain an anatomical and aesthetically satisfying shape.

Resin composite CAD/CAM blocks were launched as an alternative to ceramic and zirconia blocks. They present less hardness, less stiffness [1], and are easily fabricated [2] compared to ceramics and zirconia blocks. Furthermore, they are less brittle, easily repaired [2], and can be shaped to very thin margins with perfect biological adaptation [3,4]. Due to their reduced hardness, they are ideal materials for preservation of the opposing enamel [1].

Due to the color instability of the dual-cured resin cements [5], and with the introduction of “Deep Marginal Elevation” [6,7,8,9,10] and “cavity Design Optimization” [11] concepts, the use of pre-heated photo-polymerized resin composites as an alternative to the dual-cured resin cements was advised [12]. Photo-polymerized restorative composites reacted differently to the pre-heating treatment regarding their viscosity [13,14]. However, it was reported that pre-heating improved their margin adaptation [15]. Its advantage on the bond strength was not confirmed to be superior neither to the dual-cured resin cements [16], nor to the flowable composites [17]. In addition, decreased photo-polymerized resin composite film thickness was shown to be material-dependent [18,19,20]. The microhardness and the degree of conversion also showed controversial results, which depended on the resin composite material [21,22,23]. This was shown to be dependent on the number of the pre-heating cycles [24] and the original viscosities of the photo-polymerized composites [18].

It was noticed that pre-heating time of photo-polymerized composites varied from study to study depending on the pre-heating method [20,23,24,25,26]. It is not clear whether pre-heating of different photo-polymerized resin composites can substitute the dual-cured resin cement to bond CAD/CAM resin composite with different thicknesses. Furthermore, the definite conclusion that pre-heating of resin composites can improve the quality and the longevity of restorations was yet not confirmed [27].

The aim of this study was to evaluate the effect of: (1) CAD/CAM thickness and different composite cements on the micro-tensile bond strength (µTBS) of CAD/CAM resin composites and on the microhardness (HV) of the different composite cements; (2) the effect of the different composite cements on their film thickness (FT). The null hypotheses tested were: (1) neither the CAD/CAM thickness nor the different composite cements would have significant effect on the µTBS of CAD/CAM resin composites and the HV of the different composite cements; (2) composite cement type would also have no significant effect on the FT.

## 2. Materials and Methods

### 2.1. Materials

Resin-based hybrid CAD/CAM blocks, a dual-cured resin cement and three photo-polymerized resin composites were used as luting materials in this study. Materials (descriptions) and Lot number (Lot #), abbreviations, composition, and manufacturers are listed in Table 1.

### 2.2. Composite CAD/CAM Specimen’s Preparation

**Figure 1:**
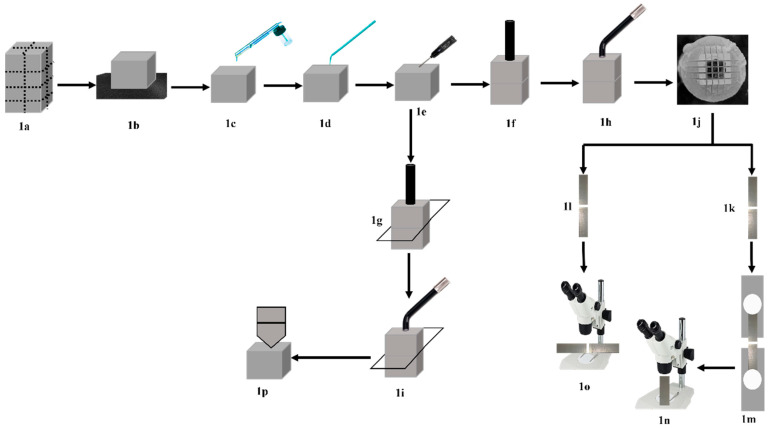
Schematic diagram for the experimental steps. (**a**) Cutting original CAD/CAM blocks into multiple small 6.8 mm × 6.8 mm × 2 mm and 6.8 mm × 6.8 mm × 4 mm blocks. (**b**) Surface grinding using #600 grit SiC papers. (**c**) Sandblasting of CAD/CAM surfaces/(**d**) Application of silane primer. (**e**) Application of luting composites. (**f**,**g**) Application of 1 Kg load for the µTBS and the HV specimens. (**h**,**i**) Photo-polymerization of luting materials. (**j**) Cutting of bonded CAD/CAM blocks in x and y directions. (**k**,**l**) Obtaining 1 mm × 1 mm bonded specimens for µTBS testing and film thickness evaluation. (**m**) µTBS testing. (**n**) Evaluation of fracture mode using a stereomicroscope. (**o**) Evaluation of film thickness using the stereomicroscope. (**p**) HV testing.

In total, nine intact CAD/CAM blocks measured 14.5 mm × 14 mm × 18 mm, which were sectioned vertically in the x and y directions and then sectioned horizontally using a low-speed diamond coated cutting disc (Isomet, Buehler Ltd., Lake, Bluff, IL, USA) under copious water irrigation to retrieve small blocks of either 6.8 mm × 6.8 mm × 2 mm or 6.8 mm × 6.8 mm × 4 mm dimensions (Figure 1a).

The surfaces of all CAD/CAM blocks (top and base blocks) were manually wet ground using #600 grit SiC paper for 10 s to create flat bonded surfaces (Figure 1b). The ground surfaces were air-abraded using 50 μm Al2O3 (MicroBlaster; bio-art, Sao Carlos, Brazil) at 0.2 MPa pressure and with an angle of 45° at 10 mm distance for 10 s (Figure 1c). The air-abraded surfaces were cleaned using sterile cotton (Cotton Buds, Cotton Stick, Egypt), saturated with 70% medical alcohol and air dried with oil/water free compressed air for 10 s, according to manufacturer instructions. Silane coupling agent (CB) was then applied using Single-Tim micro-brush (VOCO, Cuxhaven, Germany), left undisturbed for 60 s, and air dried for 10 s (Figure 1d).

### 2.3. Characterization Methods

#### 2.3.1. Micro-Tensile Bond Strength (µTBS) Testing

CAD/CAM blocks measured 6.8 mm × 6.8 mm × 2 mm (*n* = 18) and 6.8 mm × 6.8 mm × 4 mm (*n* = 18) were prepared and served as top blocks, from which photo-polymerization of the luting materials was performed. In addition, CAD/CAM blocks with (6.8 mm × 6.8 mm × 4 mm) dimensions (*n* = 36) were also prepared and served as base blocks, over which the luting materials were applied.

#### 2.3.2. Study Design

Two experimental parameters were investigated in this study, parameter 1: CAD/CAM top thickness, 2 groups (2 mm and 4 mm); parameter 2: Resin composite luting material, 6 groups (dual-cured resin cement, regular flow photo-polymerized flowable resin composite with and without preheating, regular viscosity photo-polymerized bulk fill resin composite with and without preheating, and thermo-viscous bulk fill resin composite). The total sample size was *n* = 252 rods. The sample size for each experimental group was *n* = 21 rods/group, which was retrieved from three bonded CAD/CAM blocks for each experimental group. Summary of experimental procedures is presented in Figure 1.

#### 2.3.3. Luting Material Application

Each luting resin composite was applied according to its manufacturer’s instructions. Each luting resin composite was applied over the 4 mm thick CAD/CAM base blocks (Figure 1e), followed by positioning either a 2 mm or 4 mm thick top block on top. Each cemented block was positioned in the loading device and was loaded for 10 s using 1 kg load (Figure 1f). During the loading time, excess resin cement was carefully removed from each side of the cemented blocks. Each cemented block was un-loaded, and photo-polymerization was performed from the top surface of the top block (Figure 1h) for 40 s using the LED photo-polymerization unit (Celalux 3, VOCO GmbH, Cuxhaven, Germany).

For the BFQM, the material was injected using the auto-mix tip supplied by the manufacturer. Regarding the GF and XF photo-polymerization resin composites, the caps. was placed into the applicator gun and injected over the base block. For the HGF and HXF resin composites, pre-heating of the resin composite caps. was performed using the VisCalor^®^ Dispenser (VOCO GmbH, Cuxhaven, Germany). Composite caps. of HGF and HXF resin composites were preheated at 65 °C for 70 s and the heated material immediately injected over the 4 mm thick base block. For the VCB composite, the same procedure was applied, but the heating time was adjusted to 30 s according to its manufacturer’s instructions and immediately injected as described for HGF and HXF composites.

All cemented CAD/CAM blocks received a radiant exposure of 52 J/cm^2^ (1300 mw/cm^2^ × 40 s) and a wavelength range from 450–480 nm. The light intensity was checked regularly using the built-in radiometer of the LED photopolymerization unit. All cemented blocks were stored in distilled water for 48 h at room temperature before testing.

#### 2.3.4. µ-Tensile Bond Strength Testing

After a 48 h storage period, each cemented CAD/CAM block was sectioned in x and y directions using the Isomet disc into (1 × 1) mm rods. Nine middle rods (Figure 1j) were retrieved from each CAD/CAM block (*n* = 27 rods/group). For the µTBS test, 21 rods (Figure 1k) were randomly chosen per experimental group (7 rods from each cemented block). The remaining 6 rods (2 rods from each cemented block) were used to evaluate the film thickness of each luting material (Figure 1l). For the bond strength testing, each rod was positioned on a notched metallic attachment and secured in its position using a cyanoacrylate adhesive (Pattex, Henkel AG &Co. KGaA, Düsseldorf, Germany). Each rod was pulled apart on the universal testing machine (Instron 3345, Norwood, MA, USA) at a crosshead speed of 1 mm/min (Figure 1m). The µTBS was calculated by dividing the load (Newton) over the respective cross-sectional area (mm^2^) of each rod.

All fractured top rods were evaluated for fracture mode analysis using the stereomicroscope (SMZ 745 T, Nikon, Tokyo, Japan) at 40× magnification (Figure 1n). Each failed bonded area was captured using the camera supplied by the microscope (WAT-221 S, Watec, Yamagata, Japan). The fracture modes were classified into three categories:

Category I: Adhesive mode of failure, where the fracture appeared at luting material/rod interface; Category II: Mixed mode of failure, where the fracture occurred at luting material/rod interface accompanied by part of the cement remained on the CAD/CAM rod or part of the CAD/CAM rod was fracture; Category III: Cohesive mode of failure, where the fracture occurred at the CAD/CAM rods.

### 2.4. Evaluation of Composite Cements’ Film Thickness

**Figure 2:**
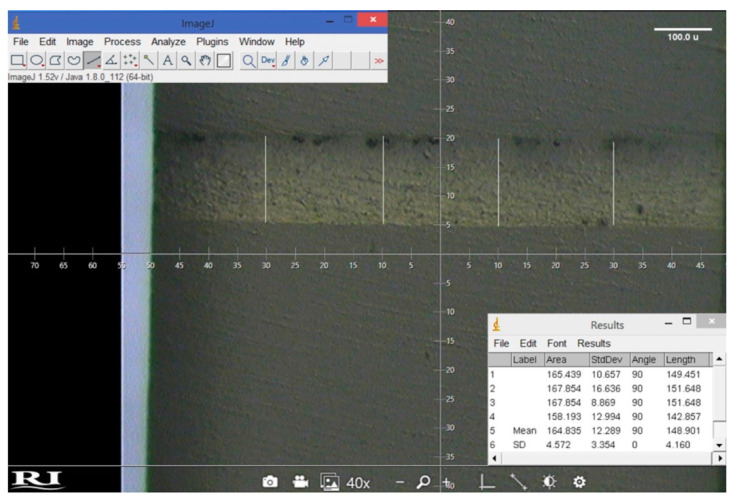
Representative stereomicroscope photo for the evaluation of film thickness (XF 4 mm group).

For each luting material, 12 (1 × 1) mm rods were chosen. The rods were randomly selected (6 rods from the 2 mm top blocks and 6 from the 4 mm top blocks) to be used for the evaluation of the film thickness. The rods were fixed over glass slides using a thin film of the cyanoacrylate adhesive in order to facilitate the polishing of the proposed examined surface. Each rod surface was polished using ultra fine polishing discs (Sof-Lex™, 3 M, ESPE, St. Paul, MN, USA) under copious water irrigation in one direction for 10 s. The film thickness was evaluated using the stereomicroscope (SMZ 745 T, Nikon, Tokyo, Japan) at 40× magnification (Figure 1o). The calibration of the microscope was performed using the stage ruler to set the scale of each photo. Each luting material area was captured using the camera supplied by the microscope (WAT-221 S, Yamagata, Japan). Each photo was analyzed using ImageJ software for Windows (ImageJ Version 1.52; Java 1.8.0_112 [46 bit], NIH, Bethesda, MA, USA, https://imagej.net/ImageJ accessed on 29 June 2021), after setting of the measuring scale. Four lines, 200 µm apart, were drawn along the luting material thickness (Figure 2). The distance between lines was standardized with the aid of the measuring scale presented in each photo. The average film thickness for each rod was calculated from the four measurements, and the overall mean of the film thickness for each luting material was calculated from the means of the 12 rods.

### 2.5. Microhardness Test

Sixty rectangular blocks of (6.8 × 6.8 × 2) mm dimensions were prepared by sectioning the original CAD/CAM blocks in the x and y directions. The blocks were divided into 12 groups (*n* = 5) according to the two experimental factors investigated, as described for the µTBS. The proposed bonded surfaces of all CAD/CAM blocks were wet ground, sandblasted, and silanated, the same as in the µTBS test. The luting materials were applied over the 2 mm thick blocks and covered with a clear Mylar strip (Matrix strips, CROSSTEX International Inc., Ranick, NY, USA). Either 4 mm or 2 mm top blocks were positioned over the Mylar strip, gently pressed, and loaded using 1 Kg load for 10 s (Figure 1g). After load removal, the luting material was photo-polymerized for 40 s from the top of the covering block using the photo-polymerization unit (Figure 1i). After photo-polymerization, the top blocks and the Mylar strips were removed, and the base blocks with their luting materials were stored dry at room temperature in light proof containers for 48 h.

After 48 h, Vickers diamond indenter was applied on the top surface of the luting material under a load of 50 g for 15 s dwell time (Figure 1p). The length of the indentation’s long diagonal was measured using a digital microhardness tester (Wilson Tukon 1102, Buehler Ltd., Lake Bluff, IL, USA) after the applied load was removed. For each block, five measurements were taken. The five measurements were averaged for each block, and the overall mean for each group was calculated from the five blocks.

### 2.6. Statistical Analysis

The effect size and the statistical power was calculated using G-Power 3.1.9.4. Statistical analysis was performed using SPSS Program for Windows (Version 21, SPSS Windows, Chicago, IL, USA). Kolmogorov-Smirnov test was used to test the normal distribution of the data. Levene’s test was used to test the homogeneity of the data. For the µTBS and microhardness (HV), two-way ANOVA was performed to analyze the effect of CAD/CAM thickness and luting composite on the µTBS and HV. For the film thickness, one-way ANOVA was performed. One-way ANOVA/Tukey’s HSD post-hoc test were used for pairwise comparison. Significance level was set at α = 0.05.

## 3. Results

### 3.1. µ-Tensile Bond Strength

The Kolmogorov-Smirnov normal distribution test for data indicated that the data was normally distributed (*p* > 0.05). The levene’s test for homogeneity of variance for the CAD/CAM thickness and the luting composites was *p* = 0.783 and *p* = 0.087, respectively. The effect size was 0.403, which indicated a strong effect size. The statistical power was 0.991, and the significant level was set as α = 0.05.

There was no pretest failed specimens, as all collected rods (21 rods/group) were tested for µTBS. Regarding the effect of resin composites (Table 2), for the 2 mm thickness, HXF revealed the highest significant µTBS among all tested resin composites *(p* < 0.05). VCB showed a statistically significant higher µTBS compared to XF and HGF (*p* < 0.05). There was no statistically significant difference between BFQM, GF, XF, and HGF (*p* > 0.05) and between BFQM, GF, and VCB (*p* > 0.05). For the 4 mm thickness, there was a statistically significant difference between BFQM and GF and between GF and HGF (*p* < 0.05). There was no statistically significant difference between BFQM, XF, HGF, HXF, and VCB and between GF, XF, HXF, and VCB (*p* > 0.05).

Regarding the effect of CAD/CAM thickness (Table 2), BFQM, HGF, and HXF showed a statistically significant difference between 2 mm and 4 mm thicknesses. There was no statistically significant difference between 2 mm and 4 mm for GF, XF, and VCB.

Two-way ANOVA revealed that parameters “CAD/CAM thickness”, “Resin cement type”, and “CAD/CAM thickness × Resin cement type” showed a significant effect on µTBS (*p* = 0.025, *p* < 0.0001, and *p* < 0.0001, respectively).

### 3.2. Fracture Mode Analysis

**Figure 3:**
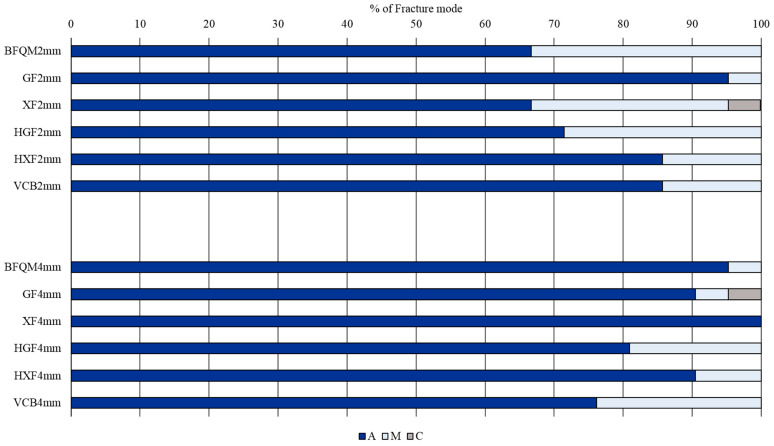
Percentages of fracture mode analysis within each CAD/CAM thickness and resin composite. A: Adhesive, M: Mixed, and C: Cohesive.

**Figure 4:**
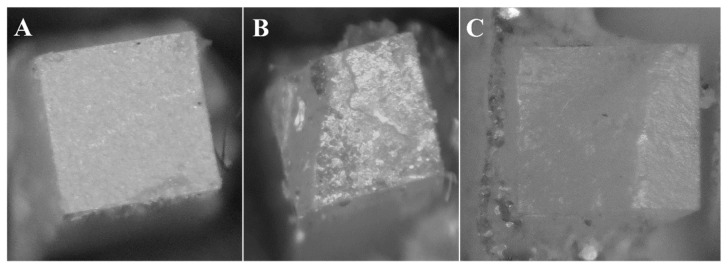
Representative stereomicroscope images for the different failure modes (40×). (**A**) Adhesive failure mode. (**B**) Mixed failure mode. (**C**) Cohesive mode of failure.

Adhesive failure was the predominate failure mode, which represents 83.73%, followed by the mixed mode, which represents 15.48%, and cohesive failure represents 0.79%. The number of specimens and the percentages of failure modes within each CAD/CAM thickness and resin composite are presented in Table 2 and Figure 3, respectively. Representative photographs for adhesive, mixed, and cohesive failure modes are presented in Figure 4A–C.

### 3.3. Resin Composite Film Thickness

For the FT, within each resin composite, statistical analysis showed that there was no statistically significant difference between 2 mm and 4 mm CAD/CAM thicknesses (*p* > 0.05). For this reason, the film thickness was statistically analyzed regardless of the thickness of the CAD/CAM resin composites, and the whole 12-rods were used to calculate the film thickness for each resin composite material. The data were statistically analyzed using one-way ANOVA and Tukey’s HSD post-hoc test. Table 3 showed that XF resin composite showed the significant highest film among all resin composites (*p <* 0.05). The VCB showed statistically significant higher FT compared to BFQM, GF, HXF, and HGF (*p* < 0.05). There was no statistically significant difference between BFQM, GF, and HXF or between GF and HGF (*p* > 0.05).

Pearson’s correlation between µTBS and FT was −0.122, which revealed no significant correlation between µTBS and FT (*p* = 0.399).

### 3.4. Microhardness

Regarding the effect of the resin composites (Table 4), for the 2 mm thickness, XF and HXF revealed the highest significant HV values, while VCB showed significant HV Values among all tested resin composites *(p* < 0.05). There was no statistically significant difference between BFQM, GF, and HGF *(p* > 0.05). For the 4 mm thickness, both XF and HXF showed the highest significant HV, while BFQM showed the lowest significant HV among all tested resin composites (*p* < 0.05). On the other hand, there was no statistically significant difference between GF, HGF, and VCB (*p* > 0.05).

Regarding the effect of CAD/CAM thickness (Table 4), there was a statistically significant difference in HV values between 2 mm and 4 mm thicknesses in all tested resin composites (*p* < 0.05).

Two-way ANOVA revealed that parameters “CAD/CAM thickness”, Resin cement type”, and “CAD/CAM thickness x Resin cement type” had a significant effect on the HV of the tested resin composites (*p* < 0.0001, *p* < 0.0001, and *p* = 0.027, respectively).

Pearson’s correlation between µTBS and HV was 0.184, which revealed no significant correlation between µTBS and HV (*p* = 0.158).

## 4. Discussion

Due to the color instability of the dual-cured resin cements [11], photo-polymerized resin composites were used to replace the dual-cured composites to bond indirect inlay/onlay restorations. The use of high-translucent CAD/CAM materials is one approach that facilitates light transmission and enhances the polymerization of the photo-polymerized composites. Pre-heating of regular-viscosity photo-polymerized composites is another approach to use them as luting materials for indirect restorations. Pre-heating reduces the viscosity, increases the flowability, and improves the adaptation of resin-based composites to cavity walls and margins [14,15,21,22,23,26,27,28,29,30,31,32,33,34], with no harmful effect, due to the elevated temperature on dental pulp [34].

The 2 mm and the 4 mm thicknesses used in this study are the most clinically relevant posterior restoration thicknesses, especially with the introduction of “deep margin elevation” and “cavity design optimization” concepts. In addition, it was reported that light transmission through the CAD/CAM materials was not detected beyond the 4 mm thickness, and the degree of light attenuation increased by increasing CAD/CAM material thickness [35].

Although the µTBS showed a high rate of pre-test failures, which was considered a disadvantage of this test [36], the µTBS was considered the most useful bond strength testing to investigate the dental materials/tooth structures bonding [37], and it is considered a reliable test to assess bonding quality for the different restorative material research [36].

The microhardness test was reported to be a tool that evaluates the degree of polymerization of materials with different specimens’ thicknesses [38]. The method determined for the microhardness test used in this study was performed to facilitate the correlation between µTBS and HV of the different composites in the same experimental conditions. Furthermore, film thickness was used in this study as an indirect method to evaluate the reduction of photo-polymerized composite viscosities and to assess their potentiality as luting materials for indirect restorations.

From the results of this study, the first null hypothesis has been rejected, as the parameters “CAD/CAM thickness” and “composite cement” showed a significant effect on the µTBS to CAD/CAM resin composite and a significant effect on HV of the different composite cements. The second null hypothesis was also dismissed, as the different composite cements showed a significant effect on their FT.

For the 2 mm CAD/CAM thickness, the results showed that *x-tra* fil composite when pre-heated yielded the higher significant µTBS. *x-tra* fil composite is a micro-hybrid composite (average filler size ≈ 10 µm), which leads to the reduction in the inorganic fillers-to-organic matrix surface area. This results in less light scattering and more light penetration through the material [39], with improvement in the material’s mechanical properties [40] and degree of conversion [41,42]. In the present study, *x-tra* fil also showed the highest significant HV values among all tested materials. The HV test can be used to determine the entire polymerization of composite specimens with certain thickness [38].

As the film thickness of x-tra fil composite reduced from 158.0 µm to 66.8 µm, which agreed with Dionysopoulos, et al. [19], it could be hypothesized that the HV values in this study may reflect the entire polymerization of the tested composite. This was an explanation, which needs further investigations. The entire polymerization of the luting resins does not depend only on the thickness of the CAD/CAM composite material, but also on material micro-structure and the out-put of the photo-polymerization unit. Grandio Blocks was reported to have higher absorption coefficient, with higher light penetration before being absorbed, which facilitates the entire photo-polymerization of the photo-polymerized composite [35]. It has been suggested that the use of photo-polymerized composites to bond CAD/CAM materials could be an alternative to the dual-cured resin cements [24,43,44,45]. By increasing the thickness of luting materials, the chance of incorporation of voids within the cured cement layer is expected to be high [46].

Furthermore, preheating of bulk-fill resin composites significantly decreases their internal voids percentage [25]. In the present study, bubbles were observed at the resin cement-CAD/CAM interface during evaluation of FT, in specimens bonded only with *x-tra* fil without preheating (Figure 2). Voids could act as stress razors that facilitate failure at lower strength [47]. The film thickness could determine the mechanical properties of the resin cement, but this showed to be dependent on the testing methodology [48]. The authors reported that, when the resin cement specimens were loaded in tension, the resin cement thickness did not significantly affect their mechanical properties, unlike the shear test, which showed reduction in the mechanical properties of specimens with reduced thicknesses. Heating was reported to enhance the adaptation of high-viscosity resin cement after 1 year of clinical evaluation [49].

The independency of the µTBS on the film thickness of the luting resin composites was observed in this study. As the CAD/CAM surface treatment, load application, application time of silane treatment, the photo-polymerization intensity, and photo-polymerization time were fixed through the whole experiment, the only uncontrolled variables in this study were the chemical composition, the filler loading percentage, and the viscosity of the different luting composites. Materials were shown to react differently upon preheating before photo-polymerization [17,18,19]. In the present study, there was statistically a significant difference between regular viscosity resin composite (XTF) and flowable composite (GF) without preheating in their FT, with XTF showing the higher FT. However, when XTF was preheated, the FT was comparable to GF without preheating. This was in agreement with the results of Tomaselli, et al. [17], which showed that pre-heating of the regular-viscosity composite reduced its thickness to the level of the FT of the flowable composite. The FT of the VCB reported in this study was higher than its FT reported by Marcondes et al. [20]. In the study of Marcondes et al., VCB was heated to 69 °C for 10 min, which was not the condition in the present study, as VCB was preheated to 65 °C for only 30 s using a VCB dispenser. Furthermore, the load applied was 150 N (≈15 Kg) in the study of Marcondes, et al., which was 15 times higher than the load applied in this study (1 Kg). 

The results of this study also demonstrated that the FT of the flowable composite (GF) showed no statistically significant difference between pre-heated and non-pre-heated groups. It was reported that the reduction in FT of a resin composite appeared to be a consequence of an increase in its flowability and subsequently a reduction in its viscosity [19]. Although this study did not assess the viscosity of the tested resin composites, the reduction in FT of the pre-heated composites could confirm the reduction in their viscosities. In this regard, it could be assumed that the results of this study agreed with the results of Loumprinis et al. [13]. They reported that the viscosity of the flowable resin composites was not affected with the increase in the temperature from 23 °C to 54 °C. Regarding *x-tra* fil composite, the results of its FT in this study disagreed with the results of Elbishari, et al. [29]. They showed that resin composites with higher filler sizes exhibited a significant increase in their viscosities, even at an elevated temperature [29]. Although the average particle size of the *x-tra* fil composite was around ≈10 µm, it showed a reduction in film thickness by ≈57%. In the study of Elbishari et al. [29], they heated resin composite to 37 °C, in contrast to this study, where resin composites were heated at 65 °C. The difference in the pre-heating temperature between the study of Elbishari et al. [29] and the present study could be the reason for such a disagreement. Based on the results of this study, although CAD/CAM thickness and resin composite luting materials showed significant effect on µTBS and HV, these results should be addressed with caution.

From the results of this study, VCB showed higher significant bond strength compared to *x-tra* fil without pre-heating. Material with large filler particle size exhibited more complex viscosity than a material with lower particle size [13]. Increasing the viscosity of the material increases the resistance of the material to flow under load [42], resulting in a higher material’s film thickness. As VCB was heated up to 65 °C before being inserted, its flow is increased, resulting in proper adaptation [50,51] and less voids formation [25]. The *x-tra* fil composite without pre-heating had a significant higher film thickness, and it was the only material that showed voids at the bonded interface. The presence of voids in specimens luted with *x-tra* fil without pre-heating may act as stress razors and facilitated the fracture of specimens in lower bond strength values compared to VCB.

The VCB showed also significant higher µTBS compared to HGF. The HGF showed the thinnest film thickness (23.4 µm) among all tested resin composites. The resin cement should be of optimum film thickness to improve its bond strength, as too thin or too thick resin cement thickness negatively affected the bond strength [47]. The lower the film thickness, the higher the stiffness of the resin cement. Resin cement should have some resiliency to withstand the pull force during bond strength testing [47]. This could explain why VCB showed higher µTBS value compared to HGF composite.

For the 4 mm thickness, GF resin composite was the only material that showed a significant lower bond strength compared to the BFQM resin cement. The GF and BFQM showed comparable FT, but the BFQM showed a significantly lower HV compared to GF. The GF resin composite is a nano-hybrid resin composite with 81 wt% filer percentage, in contrast to the BFQM, which has 70.2 wt% filler percentage. It was reported that there was a positive liner correlation between the filler loading of resin composite and its hardness properties, the higher filler loading, and the higher microhardness [52]. As the film thickness of both BFQM and GF was low, the microhardness test might reflect the entire polymerization of the material. Therefore, the higher the degree of polymerization, the expected higher microhardness of the material. Material with impaired degree of polymerization was expected to have a higher degree of linear polymers, rather than cross-linked polymers [53]. This material could be more flexible, and when loaded in tension, it might have the ability to stretch and deform before fracture [39], which in turn lead to a higher bond strength.

The non-significant differences between photo-polymerized luting composites and dual-cured resin cement raised the question: Can photo-polymerized composites replace the dual-cured resin cement to bond CAD/CAM resin composites in thicknesses up to 4 mm? According to the results of this study, the answer could be “yes”. However, the bond strength testing is not only the parameter that we can rely on to predict the quality of bonding between photo-polymerized composites and CAD/CAM materials. The bond strength of the photo-polymerized composites showed differences between the 2 mm and 4 mm thick CAD/CAM composite [16]. In the study of Goulart, et al. [16], the pre-heating of the nano-hybrid resin composite significantly improved the bond strength compared to the dual-cured resin cement, when the material was photo-polymerized through the 2 mm thick CAD/CAM composite. The opposite was detected, as only pre-heated micro-hybrid resin composite showed improvement in the bond strength when the material was photo-polymerized through a 4 mm thick indirect composite.

Surprisingly, resin composites with lower HV drop percentages, such as BFQM (6.0%), HGF (11.8%), and HXF (9.1%), showed either significant improvement in the bond strength (BFQM and HGF) or significant reduction in the bond strength (HXF). On the contrary, resin composites with higher HV drop percentages, such as GF (12.8%), XF (15.5%), and VCB (17.8%), showed an insignificant difference in their µTBS between the 2 mm and 4 mm CAD/CAM thicknesses. This can suggest that, whether the luting material bonded to either 2 mm thick or 4 mm thick CAD/CAM composite, luting composites bonding to CAD/CAM composite is shown to be material-dependent. In the present study, the bond strength of BFQM improved by ≈22% when the material was photo-polymerized through the 4 mm thickness compared to the 2 mm thickness. This was in agreement with the study of Goulart, et al. [16]. One possible explanation of this result is that the obtained HV results may suggest the entire low polymerization of the BFQM when photo-polymerized through the 4 mm thick CAD/CAM blocks. When the material was pulled during µTBS testing, the material might be expected to stretch and deform before being fractured, resulting in a higher bond strength [40].

Although the use of photo-polymerized regular viscosity, incrementally filled composites to lute CAD/CAM materials is not a new approach, the use of photo-polymerized bulk-fill and flowable composites, either with or without pre-heating, was not extensively addressed in the previous literatures. Furthermore, the evaluation of a newly introduced photo-polymerized thermo-viscus restorative bulk-fill composite as a luting material for CAD/CAM restorations was not previously evaluated. This study has several limitations: first, both µTBS and HV were evaluated after a 48 h storage time without the evaluation of the effect the long-term storage on the bond strength; second, two CAD/CAM thicknesses were only evaluated, and only one type of dual-cured resin cement was selected; third, the CAD/CAM-luting composite interface was only evaluated during the calculation of the FT of the different composites, while no further interface evaluation methods were carried out; and fourth, the bond strength was evaluated without incorporation of teeth tissues, either enamel or dentin, which eliminates the effect of tooth tissue adhesion factor in the present study. One of the limitations of this study was that a 2D model was used instead of a 3D model. 3D models, including cavity preparation, are used when testing fracture resistance of teeth or bonding to pulpal or axial walls of the cavities with different C-factors. As in different cavities, different film thicknesses were observed in different sites of the cavity and a 2D model was used for standardization. Furthermore, a material effect could not be evaluated in this study, as the thickness was not used as an independent variable along with set-up, where cement thickness represented the clinical workflow only.

Today, necessity dictates that researchers conduct more clinical studies parallel to the laboratory studied to evaluate the performance of using photo-polymerized resin composites as luting materials for indirect restorations. As photopolymerizable composites with different viscosities are delivered to clinicians, and the preheating devises are within reach, this will make clinicians’ decision to use photo-polymerized composites an alternative to the dual-cured composites, making CAD/CAM materials’ bonding more practical.

## 5. Conclusions

The following conclusions could be drawn from this study:

1. Photo-polymerized resin composites can substitute dual-cured resin cement in CAD/CAM composite bonding used in this study.
2. Luted resin composites responded differently regarding their µTBS when photo-polymerized through the 2 mm and the 4 mm CAD/CAM thicknesses, but all luted composites showed reduction in their HV when photo-polymerized through the 4 mm thickness.
3. Pre-heating improved the µTBS of the conventional bulk-fill and flowable composites when photo-polymerized through the 2 mm and 4 mm thicknesses, respectively, while no effect on HV could be observed.
4. Although, the thermo-viscous photo-polymerized bulk-fill composite showed high µTBS at 2 mm and 4 mm CAD/CAM thicknesses, a high film thickness of resin composite could be needed for its use as luting resin cement for CAD/CAM materials.

## Figures and Tables

**Table 1:** Materials (description) and lot #, abbreviations, composition, and manufacturer.

Material (Description) and Lot	Abbreviations	Composition	Manufacturer
Grandio Blocks (Resin-based hybrid CAD/CAM material, shade A2) 1950657	GB	Dimethacrylates, glass ceramics, silica Filler content: 89 wt%	VOCO GmbH Cuxhaven, Germany
BiFix QM (Dual-cured resin cement, shade universal) 2002144	BFQM	Catalyst: Dimethacrylate, BPO, silica, barium-aluminium-silicate-glass ceramics, BHT Base: Dimethacrylate, CQ, amine, silica, barium-aluminium-silicate-glass ceramics, BHT Filler content: 70.2 wt%	VOCO GmbH,
Grandio Flow (Photo-polymerized nano-hybrid regular flow resin composite, shade A2) 1948469	GF (without heating) HGF (with heating)	Bis-GMA, Bis-EMA, TEGDMA, HDDMA, CQ, Amine, BHT, SiO_2_ nano Particles, glass ceramics Filler content: 81 wt%	VOCO GmbH
*x-tra* fil (Photo-polymerized micro-hybrid regular viscosity bulk-fill resin composite, shade universal) 1951450	XF (without heating) HXF (with heating)	Bis-GMA, UDMA, TEGDMA, silicate glass. Filler content: 86 wt%	VOCO GmbH
VisCalor Bulk (Photo-polymerized thermo-viscous nano-hybrid bulk fill resin composite, shade universal) 1945171	VCB	Dimethacrylates, CQ, amine, BHT, glass ceramics, silica Filler content: 84 wt%	VOCO GmbH
Ceramic Bond (Silane coupling agent) 1949433	CB	Organic acid, 3-methacryloxypropyltrimethoxysilane, acetone	VOCO GmbH

BPO: Benzoyl peroxide, Bis-GMA: Bis-phenol A glycidyl methacrylate, UDMA: urethane dimethacrylate, TEGDMA: Triethylene glycol dimethacrylate, CQ: Campherquinone, BHT: butylated hydroxytoluene, Bis-EMA: Ethoxylated bis-phenol A dimethacrylate, HDDMA: 6-Hexanediol dimethacrylate.

**Table 2:** Means ± standard deviations in MPa (fracture modes specimen’s numbers) for the effect of CAD/CAM thickness and resin composite type on the µTBS.

	2 mm	4 mm
BFQM	35.0 ± 7.2 ^bc,^* (14/7/0)	42.9 ± 9.1 ^A,^* (20/1/0)
GF	35.7 ± 6.8 ^bc^ (20/1/0)	33.3 ± 6.3 ^B^ (19/1/1)
XF	32.3 ± 4.7 ^c^ (14/6/1)	36.6 ± 10.0 ^AB^ (21/0/0)
HGF	30.5 ± 6.3 ^c,^* (15/6/0)	41.4 ± 6.9 ^A,^* (17/4/0)
HXF	45.0 ± 8.5 ^a,^* (18/3/0)	37.3 ± 7.4 ^AB,^* (19/2/0)
VCB	38.9 ± 5.4 ^b^ (18/3/0)	38.1 ± 6.1 ^AB^ (16/5/0)

Means with same superscript small letters (^a–c^) within 2 mm thickness and with the same superscript capital letters (^A,B^) within 4 mm thickness are not statistically significant at *p* = 0.05. Asterisks denote (*) a statistically significant difference between 2 mm and 4 mm within each luting composite. (a/b/c) represents the adhesive/mixed/cohesive specimen’s number within each resin composite.

**Table 3:** Means ± standard deviations in µm for the effect of resin composite type on film thickness of the different resin composites.

BFQM	GF	XF	HGF	HXF	VCB
64.4 ± 14.6 C	46.1 ± 8.5 CD	158.0 ± 26.8 A	23.4 ± 3.8 D	66.8 ± 13.1 C	121.8 ± 28.5 B

Means with same capital letters (A–D) are not statistically significant at *p* = 0.05.

**Table 4:** Means ± standard deviations for the effect of CAD/CAM thickness and resin composite type on HV (Kgf/mm^2^) of the different resin composites.

	2 mm	4 mm
BFQM	57.9 ± 2.9 ^c,^*	45.4 ± 2.2 ^C,^*
GF	60.3 ± 2.8 ^c,^*	52.6 ± 4.4 ^B,^*
XF	80.7 ± 3.6 ^a,^*	68.2 ± 3.5 ^A,^*
HGF	58.5 ± 1.7 ^c,^*	51.6 ± 1.1 ^B,^*
HXF	79.4 ± 1.7 ^a,^*	72.2 ± 2.2 ^A,^*
VCB	66.5 ± 1.8 ^b,^*	54.6 ± 1.6 ^B,^*

Means with same superscript small letters (^a–c^) within 2 mm thickness and with same superscript capital letters (^A–C^) within 4 mm thickness are not statistically significant at *p* = 0.05. Asterisks denote (*) the statistically significant difference between 2 mm and 4 mm within each luting composite.
